# Immunization as an entry point for primary health care and beyond healthcare interventions—process and insights from an integrated approach in Lebanon

**DOI:** 10.3389/frhs.2023.1251775

**Published:** 2023-10-27

**Authors:** Bhrigu Kapuria, Randa S. Hamadeh, Farah Mazloum, Kassem Chaalan, Kyaw Aung, Ettie Higgins, Wafaa Kanaan, Tatiana Tohme, Doaa Kamal, Christina E. Khoury, Sabin Syed

**Affiliations:** ^1^ UNICEF Lebanon Country Office, Beirut, Lebanon; ^2^ Social Health Services & Primary Health Care Department, Ministry of Public Health, Beirut, Lebanon; ^3^ DRR, Lebanese Red Cross, Beirut, Lebanon

**Keywords:** immunization, integration, outreach, comprehensive healthcare, primary healthcare immunization, primary healthcare

## Abstract

Integrated healthcare systems are continually pitched as major contributors towards better distribution of health outcomes and enhanced well-being. Under emergency conditions, integrated healthcare services can guarantee better access to the target population. In recent years, several crises, i.e., economic collapse, the fuel crisis, the Beirut blast, a large refugee population, and the COVID-19 pandemic, in Lebanon have led to a major shift in the health-seeking behavior of the communities, with preventive services being downprioritized despite being available and curative healthcare services being sought out as late as possible. An extensive drop in immunization coverage and an overstretched public health system presents the risk of Vaccine-Preventable Disease outbreaks and urgent intervention is needed to bridge the immunity gap. The Ministry of Public Health, Lebanon, and UNICEF Lebanon successfully demonstrated the use of an immunization platform as an entry point to reach communities for service delivery, identification and referral, screening, awareness generation, and a host of other services that can be copied for other programs including but not limited to those for Maternal and Child health, nutrition, early childhood development, COVID-19, children with disabilities, social protection, education, health emergencies like cholera, etc., and these can provide bi-directional support to each other. UNICEF along with the MoPH (Ministry of Public Health) has been working towards reaching the most vulnerable population with a bouquet of services through existing immunization touchpoints for favorable healthcare outcomes.

## 1. Introduction

Health systems combine both integrated and individual interventions. Integration is best seen as a continuum rather than as the two extremes of integrated or not integrated. Routine immunization has a long history of integration with multiple programs, like vitamin A supplementation, growth monitoring, deworming, or insecticide-treated bed nets (1). The importance of integration, both in health systems in general and within immunization programs more specifically, has been continually growing, and this is reflected in a broad range of global policies and strategies.

There has been much interest in using immunization as a platform for other interventions since immunization coverage is relatively high in most countries compared to other interventions along the continuum of care (1). In addition, childhood immunization organizes regular immunization contact at set intervals, such as five contact moments in the first year of life and additional contact during the second year of life, school age, and adolescence depending on the national immunization schedule. Immunization is a global health and development success story, saving millions of lives every year. Immunization is the foundation of the primary health care system. However, it is important that the scheduling of integrated services is assessed to determine their feasibility based on the human and material resources needed vs. those available.

A life course approach to immunization can facilitate integration opportunities. For example, the delivery of a birth dose of the Hepatitis B vaccine could be provided alongside other postnatal care and be used as a key advocacy opportunity to inform the parents about the national immunization schedule and provide them with a home-based record for their child. The need to identify all newborns in the community to provide the birth dose vaccinations could also strengthen systems for civil registration and vital statistics, which could in turn improve the denominators used to monitor immunization coverage rates.

Globally there have been multiple efforts to sync immunization services with other country-specific domains or areas of concern in many countries, such as the United States Agency for International Development's Maternal and Child Survival Program (MCSP) supported the Liberia Ministry of Health to scale up integrated family planning and immunization services as part of a broader service delivery and health systems recovery program after the Ebola epidemic (2). The Government of Malawi's *Health Sector Strategic Plan II* highlights the importance of service integration and systematically implemented integrated family planning and immunization services in all health facilities and associated community sites in the Ntchisi and Dowa districts during the period June 2016–September 2017; results indicated statistically significant increases in family planning users and shifts in the use of family planning services from health facilities to community sites (3). In an assessment in Rwanda, 98% of women interviewed supported the idea of integrating family planning service components into infant immunization services (4). Additionally, a study conducted in two northwest Ethiopian districts and another study conducted with survey data from Ethiopia, Malawi, and Nigeria found an association between contraceptive use and child immunization (5, 6). In Lao PDR, the use of this integrated approach compared with the implementation of the vertical deworming campaign alone allowed a reduction of the individual cost of deworming by 10 times (from US$0.23 in the vertical deworming campaign to US$0.03 in the integrated campaign). The burden on health workers by the integration process was perceived as minimal and manageable. Moreover, delivery of anthelminthic drugs during immunization campaigns enabled campaign teams to observe drug intake directly, which ensured safety (7).

The Global Routine Immunization Strategies and Practices (GRISP) document includes “integrating the routine immunization program through comprehensive approaches and joint service delivery” (8) as one of the key strategies to maximize the reach of routine immunization and mentions placing vaccines into the context of comprehensive approaches to disease control, delivering other key preventive maternal and child health interventions during vaccination visits where appropriate and starting immunization program tracking with pregnant women and during antenatal care, as key interventions. The provision of outreach services is advocated to be a central strategy in countries where fixed sites are inadequate for regular provision of preventive health services to all targeted individuals (9).

Immunization is the most strategic component of primary health care aimed at preventing diseases through primary healthcare (10), and it is also one of the best health investments money can buy. Vaccines are also critical to the prevention and control of infectious disease outbreaks. Researchers from Gavi and the Federal University of Pelotas, Brazil, investigated the overlap between not being vaccinated with routine immunizations and failing to receive other primary healthcare services. They analyzed data from more than 200,000 infants aged 12 to 23 months between 2010 and 2019 from 92 LMICs. They found that unvaccinated children and their mothers were systematically less likely to receive other primary healthcare interventions, particularly for antenatal visits, and access institutional delivery. Families whose children have no access to vaccination are missing out on crucial primary healthcare services that affect both mothers and children and offer an opportunity for integrated service delivery to reduce inequity. Identifying and reaching zero-dose children is likely to be critical to tapping into families who are missing out on important primary healthcare interventions (11).

Integration of CoVID-19 Vaccination delivery into routine immunization services has yielded favorable outcomes across many countries. The initial arrangement of COVID-19 vaccination campaigns, i.e., mass-level vaccinations, was deemed a non-sustainable model in terms of finances as well as human resources; there was also an increasing risk of adverse effects on routine immunization services, leading to increased interest from other countries and efforts toward integration of the COVID-19 immunization services with the routine immunization.

Outreach services (an extension of facility-based primary care services used to reach the underserved), campaigns, and outbreak responses are key interventions deployed to increase immunization coverage, reduce the risk of outbreaks, and address the barrier to access. Outreach often plays an important role in systematically delivering immunization services to a large proportion of the population—in some cases reaching >50% of the target population. In addition to providing routine immunizations, outreach sessions present opportunities to provide women, children, and their families with other vital interventions, such as vitamin A supplementation, deworming tablets, and insecticide-treated nets (ITNs). “Reaching every district” (RED) is a strategy to achieve the goal of 80% immunization coverage in all districts and 90% nationally in the WHO member states. Outreach sessions have been recognized as a key activity for WHO's RED strategy. Outreach sessions, especially mobile teams, present opportunities to provide other interventions along with immunization (12).

Lebanon has traditionally been an upper middle-income country and has had a significant share of the private healthcare sector for both preventive as well as curative services but is now facing a cascade of crises. The Lebanon financial and economic crisis is likely to rank in the top 10, possibly top 3, most severe crises globally since the mid-19th century. This is a conclusion of the Spring 2021 Lebanon Economic Monitor (LEM) in which the Lebanon crisis is contrasted with the most severe global crises as observed by Reinhart and Rogoff (2014) (13) over the 1857–2013 period. In July 2022, the World Bank reclassified Lebanon as a lower-middle income country (LMIC), down from an upper-middle income country (UMIC) (14). Amid its worst socioeconomic crisis in decades, Lebanon has a unique situation in hosting the highest number of refugees per capita worldwide. The Government estimates that there are 1.5 million Syrian refugees and 13,715 refugees of other nationalities; 90% of Syrian refugees are living in extreme poverty (15).

Lebanon's once robust healthcare system has buckled under the weight of the economic collapse and COVID-19. Hundreds of healthcare workers have fled the country in “a mass exodus” unable to withstand the chronic shortages of staff, basic medical supplies, and pay. The August 2020 explosion damaged 292 health facilities. As the economy has deteriorated and poverty has risen, private health care has become unaffordable for many, increasing the strain on the depleted public health sector. The healthcare system has been battling with poor healthcare outcomes since 2020, and Lebanon is witnessing an extensive drop in immunization coverage with only 67% of children receiving the initial doses of basic vaccination. Various factors contribute to the drop in immunization coverage with non-affordability of the private sector, challenges in accessing public health facilities due to rising transportation cost, and the shrinking value of income coupled with low priority at the family level to spend precious and limited time and resources on getting a child vaccinated leading to delays and postponement.

Furthermore, the IPC (Integrated food security Phase Classification) Acute Food Insecurity analysis carried out in all 26 districts showed that 33% of the Lebanese resident population and 46% of the Syrian refugee population (16) were estimated to be in IPC Phase 3 (Crisis) or above, requiring urgent humanitarian action to reduce food gaps, protect and restore livelihoods, and prevent acute malnutrition. The rising food insecurity is a major threat to the nutritional status of the country. From being the first country to achieve SDG goals for the last two decades, Lebanon had been known to maintain stable maternal mortality rates, however maternal mortality rates tripled between 2019 and 2021 from 13.9/1,000 to 47.6/1,000**.** Likewise a three-times rise in neonatal deaths, especially among Syrian refugees, e within the span of 2 years (2019–2021) has been observed. All the above factors highlight a need for multi-dimensional integration of methods to address various ongoing as well as underlying crises.

UNICEF, along with the MoPH and locally active NGOs, is working towards interventions to not only ensure uptake of immunization but also explore arrays of integration—to use the opportunities of immunization interventions as a platform to provide impetus to efforts directed at multi-dimensional health and well-being efforts for children. Immunization has a long history of integration with a broad range of other health services delivered using both routine and campaign-based delivery strategies.

## 2. Materials and methods

Post identifications of factors like economic and fuel crises, which were the major barriers to seeking immunization, it was deemed appropriate to move from the existing facility-based approach towards the community-based service through outreach sessions. The transportation affordability was a major cause of the continuously dropping rate of immunization, leading to a mammoth rise in unvaccinated as well as under-vaccinated children in the country. Following the COVID-19 pandemic, 1 in every 3 children born are estimated to be missing either one or more vaccines, while 1 in every 10 children are part of a zero dose children cohort. Nearly one-third of the children are subjected to a riskof measles, and because of the overall compromised scenario, Lebanon has faced its first devastating outbreak of Cholera in 29 years. Cumulatively, the country stands vulnerable to multiple VPD (Vaccine Preventable Disease) outbreaks, including polio, measles, etc.

To minimize the risk of VPD outbreaks and make immunization more accessible, the MoPH, UNICEF, and the Lebanese Red Cross (LRC) have joined hands to identify high-risk areas, i.e., areas with a high number of missed or dropped out children. Nine priority districts were identified initially in early 2022; to further intensify the efforts in consultation with district officers, pockets with more urgent need of interventions were identified within these nine districts to ensure that the immunization services are delivered closer to the most vulnerable populations.

Before the commencement of each outreach session, LRC through its network of Volunteers on a national scale, would create awareness of and involvement in activities through house-to-house/door-to-door visits by informing and mobilizing parents and engaging with community leaders, municipalities, schools, religious leaders, etc.

In order to utilize immunization as an entry point for the delivery of a diverse range of healthcare and additional services, a multifaceted coordination strategy was put in place under the aegis of the Ministry of Public Health (MoPH). This involved formalizing a technical and financial collaborative agreement between UNICEF and the Lebanese Red Cross (LRC). Additionally, the MoPH spearheaded the creation of a working group comprising representatives from each participating organization. This group was involved in the ongoing monitoring of the initiative's implementation, identification of operational challenges, and the generation of monthly progress reports. Adaptation plans were formulated as needed based on these assessments. To further support the endeavor, UNICEF conducted periodic field visits to furnish on-site assistance as necessary. This systematic approach was designed to ensure the seamless integration and success of the initiative.

In the development of the integration plan, the Ministry of Public Health (MoPH) collaborated closely with UNICEF and the Lebanese Red Cross (LRC), as UNICEF is the lead partner to MoPH on immunization with a focus on strengthening primary healthcare services in Lebanon. Additionally, the organization leads the education sector in Lebanon and implements various initiatives for child protection, WASH, and other interventions around mothers and children in Lebanon. On the other hand, the LRC is distinguished as one of Lebanon's most trusted non-governmental organizations and is renowned for its emergency response capabilities. With a robust network of over 12,000 community-based volunteers, the LRC serves as the first responders in times of emergencies. Moreover, the organization also operates the majority of the ambulance services in the country, further solidifying its credibility among local communities. Therefore, the collaborative efforts leveraged the unique strengths of each partner to develop a comprehensive approach to integration of healthcare and other services in Lebanon.

The main aim of using ‘Immunization as an Entry Point (IaEP)’ during these visits was to utilize the available resources to create awareness and linkages to other healthcare services than immunization. This was done by providing integrated and age-appropriate MNCH messages beyond mobilization for the upcoming immunization session. COVID-19- and Cholera prevention-related messaging and promotion of COVID-19 vaccination became an integral part of the entire intervention as standalone COVID-19 interventions were not being received well by the community. The same visits were also used during the cholera outbreak to sensitize the community on prevention, early detection, and care seeking behavior.

The approach was to use ‘Immunization as an Entry Point (IaEP)’ for other health and beyond-health interventions during the time available when parents/caregivers and children were at a session site. The pre-vaccination waiting time, during immunization time, and post-immunization observation time were key opportunities identified to provide other services and open doorways for referral and linkages. In a regular outreach session, the pre-vaccination waiting time averaged 15 min followed by 5–10 min of time for vaccination (depending on the number of vaccines to be administered) and followed by a post-vaccination observation time ranging 20–30 min. Overall, every parent/caregiver was at the session site for 45–60 min, and we managed to use this time for IaEP. It is important to state that the intervention was designed with a clear understanding that all services of PHC or comprehensive interventions of each program cannot be provided at the immunization session, and the intention was only to create ‘Entry Points’ so that linkage to other services can be established. It is essential to note that ensuring parents/caregivers stay at a session beyond 45–60 min becomes challenging and leads to a drop in participation. Another important fact to note is that post-vaccination, children and young infants often cry, and parents/caregivers are engaged in calming the child. Therefore, a delicate balance of limited information, messaging, and interventions needs to be planned to ensure effective uptake. We used this opportunity to discuss and screen children and identify challenges in the domains of education, malnutrition, and maternal and child health and finally link the identified beneficiaries to relevant departments and child welfare schemes. UNICEF has a child-centric approach and multiple levels of intervention for various domains ranging from health to education, child and social protection, disability, and gender inclusion, and it has become an effective platform for integration and linkage.

Integration of the other services with immunization can help to address the major challenges faced by communities, i.e., access to health facilities due to the total absence of public transport and the high cost of travel within the context of shrinking income. Also, the rising trust in the public routine immunization system of parents can further add impetus to other initiatives adopted by these families if these services are advocated for through immunization service delivery platforms. The domains mentioned below were explored for possible integration with the immunization services either for delivery of service, creating awareness advocacy, or for screening purposes.

### 2.1. Registration of missed children

Lebanon maintains digital health records for every child seeking services from the public health system. The immunization status of every child is entered in the PHENICS (Primary HealthCare Network Information and Communication System) or its mobile program MERA (Mobile EPI Registry Application). During outreach intervention, all children missing from the database were entered and their vaccination records were updated. Once the child is part of the database, it becomes easier for the PHCC to follow up with parents for the next vaccination, identify drop-out cases, and plan interventions. The MoPH and UNICEF also developed a mobile application for parents called ’Sohatona’ to track the vaccination status of their children, and this is linked to a PHENICS/MERA database. During the vaccination session, parents were informed about the Sohatona application and were updated about the PHCC network, the nearest PHCC, and the services offered. All PHCC-related details, including geo-locations on the map, are also available in the Sohatona application for easy navigation by parents/caregivers.

### 2.2. Malnutrition

Healthcare workers and volunteers involved in outreach immunization activities were trained in using MUAC (Mid-Upper Arm Circumference) tape to screen children between 6 and 59 months of age. During the screening process, children identified as malnourished were referred to the ongoing malnutrition management program by the MoPH and supported by UNICEF. The screening was done when the child reached the session site and occurred during the process of registration. The result of the screening was added to the individual digital database along with vaccination details using the MERA application. Children were also provided with micronutrient supplements and parents/caregivers were oriented on frequency and administration.

### 2.3. COVID-19

With the multiple ongoing crises, discussions related to COVID-19 in 2022–23 became a low priority for individuals and communities and attempts to create exclusive COVID-19-related interventions, including COVID-19 vaccination, were not gaining any traction from the community. We integrated COVID-19 prevention with motivation for getting a COVID-19 vaccine and facilitating registration and referrals for vaccination, and this became a part of the integrated outreach intervention. This led to the preservation of pandemic-related intervention, mobilizing the community for the purpose of COVID-19 vaccination and better acceptance of continuous dialogue around COVID-19 as part of the overall preventive-promotive health discussion.

### 2.4. Education

One of the major priorities and commitments for UNICEF is school education for all children. The outreach immunization program provided a noteworthy prospect to identify the out-of-school children when they come for vaccination thus acting as an ‘Entry Point’. Identification of areas with large numbers of out-of-school children is also supported in the prioritization of education-related interventions. The out-of-school children were connected to various ongoing interventions by UNICEF and other stakeholders to facilitate school enrolment and other interventions to facilitate children in need of support.

### 2.5. Child welfare

UNICEF closely works with communities to ensure that the most vulnerable children are supported and protected. UNICEF's cash incentive scheme “Haddi” for the most vulnerable children became a proxy indicator to check if outreach sessions are reaching the most vulnerable areas while ensuring the most marginalized children are vaccinated and protected from deadly disease. Families enrolled under the “Haddi” scheme were informed about the upcoming sessions in their area and were motivated to take their children for vaccination thus protecting them from diseases.

#### 2.5.1. Children with disability/es

Children with any form of disability often face challenges in getting vaccinated due to issues related to access as well as prioritization. The integrated outreach intervention focused on identifying children with special needs and motivating their parents to get them vaccinated. The activity supported reaching out to a large number of missing children with disabilities. Although the intervention did not create a separate indicator to document the number of “children with special needs” getting vaccinated, the on-ground implementation indicated successful outreach to many such children. Immunization can also become an entry point to link children with special needs to relevant intervention and support schemes by the Government and partners.

### 2.6. Maternal and child health

Parents and caregivers attending immunization sessions were provided with context and audience-specific integrated messaging around breastfeeding, weaning, childcare, identification and timely health seeking for basic child health disease, etc. This integrated messaging focused on updating mothers with age-specific preventive and promotive behaviors for the betterment of their own health as well as that of their children, key awareness generation, and information related to health-seeking behaviors for PHCs.

### 2.7. Management of other disease outbreaks

During the intervention, Lebanon witnessed a rapidly spreading Cholera outbreak 29 years after the last reported case. Immunization sessions were used as an entry point to address the cholera outbreak as well. While engaging with the community for immunization, awareness generation on cholera was incorporated to make the community aware of prevention, symptoms, and the need to seek immediate care. The community engagement team also carried ORS sachet and an additional screening questionnaire was added to identify any family member suffering from symptoms of Acute Watery Diarrhea (AWD). For any symptomatic individual, five ORS sachets were provided, and family members were oriented on the creation and use of ORS solutions along with information related to health facilities managing cholera cases. The information on potential cases was also shared with the surveillance and health teams for follow-up.

The integrated approach identifies indicators for each of the domain areas, and the absolute numbers of beneficiaries reached against each indicator, and this will support the quantification of the efforts made to provide comprehensive services to children, using immunization as an entry point.

## 3. Results

The efforts to reach and vaccinate missed children resulted in vaccination of >200,000 children and adolescents from Dec 2021 to March 2023, during the phased scale-up approach. In addition, as reflected in Table 1, the supplementary activities added after September 2022 resulted in reaching out to nearly 798,125 caregivers for MNCH-related services, making this the domain's biggest beneficiary from the integration efforts. Other noteworthy results were reaching 363,926 individuals with cholera prevention information, and 322,284 individuals were reached for COVID-19-related messages. A lot of effort was put into integrating education, child protection, and nutrition linkages, and analysis shows that although considerable reach could be attained through an integration approach, more strategies and implementation methodologies can be looked into for further reach.

**Table 1:** Outcome of integration activities.

Domain	Indicators	Number
Immunization	Number of missed children and adolescents reached with vaccination	2,24,574
	Number of missed children receiving first dose of DPT-containing vaccine as per the national dropout protocol	25,219
	Number of missed children receiving the first dose of Measles vaccine	14,070
	Number of children receiving their first dose of IPV (Injectable Polio Vaccine)	11,933
Nutrition	Children screened with MUAC tape	87,724
	Children identified with moderate malnutrition and referred to Health Facilities for management of malnutrition	297
	Children identified with severe malnutrition and referred to Health Facilities for management of malnutrition	42
	Children provided withmicronutrient supplements	5,691
Education	Identified and referred out of school children	2,482
Child protection	Number of vulnerable children enrolled under cash incentive scheme reached and vaccinated	6,482
Maternal and child health	Number of parents/caregivers reached with integrated MNCH messaging	7,98,125
COVID-19	Number of individual reached with awareness generation related to prevention of COVID-19 and promotion of COVID-19 vaccination	3,22,284
Cholera outbreak	Number of individuals reached with information related to Cholera prevention and control	3,63,926

## 4. Discussion

Compared to Lebanon, many countries face the challenge of limited healthcare resources, and many policy- and decision-makers are faced with decisions on where and how to prioritize resources for certain healthcare services and technologies for different groups in an equitable manner to achieve more effective care. In these scenarios, using an immunization platform to develop a minimum package of essential healthcare and child welfare services, ensuring availability and accessibility for the beneficiaries, can contribute to improving the overall childcare spectrum.

Providing additional integrated services (such as bed nets/hygiene kits or other relevant commodities) may provide increased motivation to caregivers to fully immunize their children. Integrated service delivery may help to increase efficiency as operational costs are shared across programs and could therefore contribute to their long-term sustainability.

Improving links between immunization programs and other services (nutrition, education, ECD, MNCH) can help ensure that each contact with a child for immunization acts as an ‘entry point’ for multiple other interventions in health and beyond health domains. Similarly, cross-linkage between other interventions interacting with children and adolescents can be used to screen and improve immunization coverage, especially for booster dose vaccination.

Integrated service delivery may reduce the costs of reaching hard-to-reach populations in the future. The global approach of “life-course vaccination” and with introduction of new vaccines targeting extended age groups, such as HPV vaccine for adolescents, provide an opportunity to reach these populations with broader health interventions (such as deworming, sexual, and reproductive health education, etc.), which may, in turn, also increase demand for vaccination. Ongoing scientific advancement in new ways of vaccine delivery, for example, microarray patches or compact prefilled auto-disable devices, will simplify the delivery of immunizations in the future and may further facilitate integrated service delivery at the facility and community level.

It is important to note that under the concept of “integration”, services cannot and should not be packed in one service delivery model or intervention, especially at community-level intervention. Every intervention has some unique pre-requisite to successfully reach the desired output, however, attempts should be made to create inter-linkages and gateways to connect the beneficiary with a host of other interventions benefiting children, adolescents, women, and the community at large. This approach supports the identification of individuals and populations in need of specific services and facilitates better distribution of limited resources to already identified individuals rather than starting afresh, e.g., screening children for malnutrition during the immunization session supports identification of malnourished children and directly connects them with nutrition-led intervention to treat these children without additional financial investment in screening thus effectively utilizing nutrition funding. In addition, geographies reporting a high number of malnourished children could be a focus area for detailed nutrition intervention.

Lack of integration among existing healthcare systems may serve as a major limiting factor in future pandemics, as an unintegrated system does not have the capacity to prevent cases from spreading rapidly and converting into an outbreak or potential epidemic or even pandemic.^i^ Well-integrated healthcare systems are people-centric responsive units that can be easily leveraged for better outcomes during future pandemics through expanded surveillance through existing systems using digital technology to identify and communicate epidemiological changes and trends. Integration can be key for better outbreak responses in the future through leveraging of existing linkages among different healthcare delivery systems. With immunization programs being one of the most cost-effective public health interventions reaching a maximum number of people and families at the community level and vaccination now being an integral component of any future pandemic response, using immunization as an entry point for regular public health programs as well as an effective route for pandemic/epidemic/emergency responses is well demonstrated through intervention.

Successful integration of other health and non-health interventions with immunization requires a series of carefully planned and implemented steps, including “selecting interventions that can be feasibly integrated; instituting intersectoral coordination at all program levels; exploring funding for integrated interventions; conducting joint training and supervision of health workers and program managers; ensuring the participation of community based organizations, leaders, and volunteers; and establishing a robust monitoring and review mechanism”.

## Data Availability

The raw data supporting the conclusions of this article will be made available by the authors, without undue reservation.
